# Prevalence and associated risk factors for chronic kidney disease in the elderly physically disabled population in Shanghai, China: a cross-sectional study

**DOI:** 10.1186/s12889-023-16455-4

**Published:** 2023-10-12

**Authors:** Hengjing Wu, Yao Li, Longbing Ren, Jue Li, Yiyan Wang, Chenghua Jiang, Jing Wu

**Affiliations:** 1 Clinical Center for Intelligent Rehabilitation Research, Shanghai YangZhi Rehabilitation Hospital (Shanghai Sunshine Rehabilitation Center), School of Medicine, Tongji University, Shanghai, 201619 China; 2 China Center for Health Development Studies, Peking University, Beijing, 100091 China; 3 Department of Fundamental Nursing, School of Nursing, Shanghai University of Traditional Chinese Medicine, Shanghai, 201203 China

**Keywords:** Risk factor, Chronic kidney disease, Stage 3–5, Disability level, Older adults

## Abstract

**Background:**

The global prevalence of chronic kidney disease (CKD) in the general population is relatively clear. Our previous study showed that elderly individuals who are physically disabled are more likely to experience kidney function impairment, and the main purpose of this study was to determine the prevalence and risk factors associated with CKD in elderly patients with physical disabilities.

**Methods:**

A total of 2679 elderly individuals with physical disabilities from the 2018 Shanghai Disability Health Survey were screened to calculate the prevalence of CKD. Multiple logistic regression was performed to identify the factors associated with CKD. Detailed subgroup analyses of disability level were also conducted.

**Results:**

We confirmed CKD in 287 of 2679 (10.7%) participants. Female sex, age, history of hypertension, red blood cell count, albumin, urea, and uric acid (UA) were independently correlated with CKD. Age and UA abnormalities were common risk factors for different levels of disabilities.

**Conclusion:**

The prevalence of CKD is higher in the mild level of older physically handicapped individuals. Age and the level of UA should also be considered in this population. The preventive strategies for patients with two levels of elderly disability should have different focuses.

**Supplementary Information:**

The online version contains supplementary material available at 10.1186/s12889-023-16455-4.

## Background

Chronic kidney disease (CKD) has become a major global public health problem with a substantial economic burden. Globally, in 2017, there were 697.5 million cases (the prevalence was estimated as 9.1%) of CKD, and China had the highest number of CKD patients (132.3 million cases and 9.5% prevalence) [1]. Meanwhile, CKD ranked 17th in the list of causes of total deaths worldwide in 1990 and rose to 12th in 2017 [1]. CKD is widely regarded as a disease multiplier because it increases the risk of other common related complications, including acute kidney injury [2], mineral and bone diseases [3], anemia [4], and death from all causes and cardiovascular diseases [5]. In both developing and developed countries, less than 10% of patients with CKD are aware of their disease because most patients have an insidious onset and have no symptoms in the early stage [1]. Patients with stages 3–5 CKD account for nearly half of CKD cases worldwide [1], and most of them gradually develop end-stage renal disease (ESRD) and require dialysis or renal transplantation due to the irreversible nature of CKD stages 3–5 [6].

The rapid increase in risk factors such as aging, diabetes, hypertension, and obesity will lead to an even heavier burden of a higher prevalence of CKD in the coming decades [7, 8]. The prevalence of CKD stages 3–5 in the elderly 60 years and older was 4.9% in China [9], while the rate of CKD stages 1–5 among the same age group is 18.2% in the UK [10]. However, the link between some laboratory test variables and CKD is controversial, such as albumin (Alb) [11, 12] and uric acid (UA) [13–15]. Furthermore, impaired physical performance and disability are common in older adults with CKD [16, 17], so the current elderly population for prevention and treatment of CKD should also emphasize people with disabilities. The onset and general patterns of elderly disabled patients with chronic diseases are usually different from those of their healthy peers [18]. To date, epidemiological studies of CKD in the elderly general population are relatively abundant, and less attention has been given to the elderly disabled population [16]. Relevant data on the number of elderly people with disabilities are limited, but over 1 billion people have disabilities worldwide (approximately 15.6%), of whom approximately 110–190 million suffer from severe functional impairments [19]. In addition, the number of people with disabilities in China has reached 85 million, accounting for 6.21% of the total population [20]. Our previous study showed that patients with elderly physical disabilities have a higher risk of hypertension, diabetes, and other chronic diseases, which are more likely to cause kidney function impairment[21].

Therefore, we conducted a cross-sectional study to identify the prevalence of and potential risk factors for CKD stages 3–5 in the elderly (over 60 years) physically disabled population in Shanghai, China.

## Methods

### Study population

**Fig. 1:**
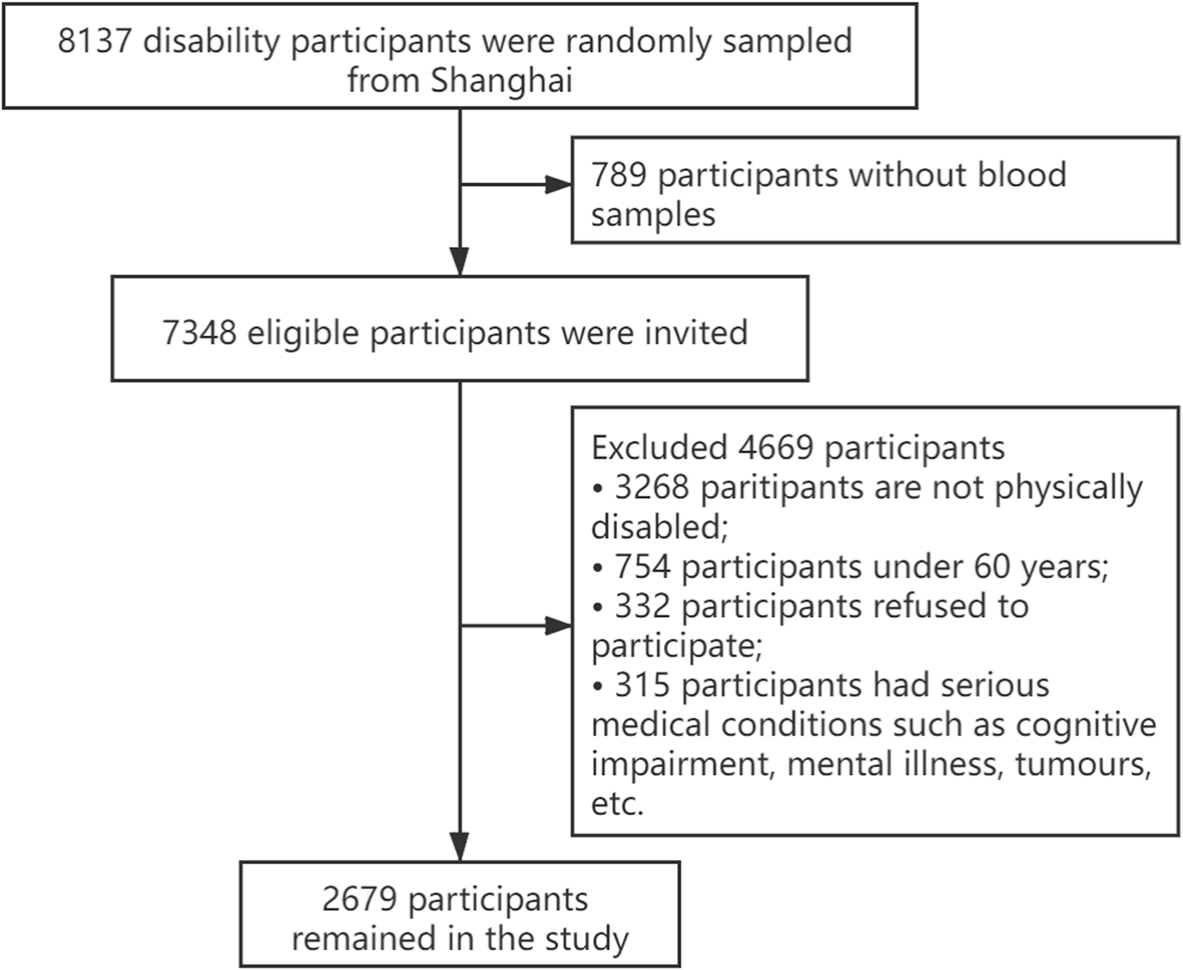
Flowchart of study population recruitment

The Shanghai Disability Health Survey (SDHS) is a nonprofit, disability-focused survey project initiated by the Shanghai Disabled Persons' Federation. The SDHS provides free health examination services to disabled people in Shanghai through designated medical institutions every year. Shanghai YangZhi Rehabilitation Hospital (Shanghai Sunshine Rehabilitation Center) is one of the designated medical and health care institutions for the disabled by the Shanghai Disabled Persons’ Federation. A total of 8,137 potential disabled participants were recruited for the SDHS project at Shanghai Yangzhi Rehabilitation Centre from February 2018 to December 2018 through community billboards and mobile short messages. This study selected 2,679 participants with disabilities based on the exclusion criteria: 1, nonphysically disabled participants; 2, under 60 years old; and 3, presence of severe mental and major organ diseases, such as a tumour and dementia.; 4, failure to complete blood biochemical tests; and 5, refusal to participate in the study, as shown in Fig. 1.

### Measurements

All participants had a medical and functional assessment by a medical professional. The health examination includes questionnaires, physical examinations, and laboratory tests. Education and medical history were based on the participants' self-report. Blood pressure (BP) was measured using a cuffed electronic sphygmomanometer (Omron Corp., Tokyo, Japan). After participants sat still for 5 min, BP was measured twice on their left upper arm at 10-min intervals, and the two recorded values were averaged. For participants who lacked upper limbs, the physician took BP measurements from both limbs (ankle – posterior tibial artery) in their prone position and used the limb with the highest reading. In this study, a total of 11 participants underwent leg BP measurements, and the results were transformed by a formula according to the recommendation of the literature [22, 23] that was used to ensure consistency with arm measurements. Body mass index (BMI) was calculated according to the formula weight (kg)/(height (m)^2^), with the height measured in the supine position for those unable to stand.

Laboratory tests included plasma red blood cell count (RBC), white blood cell count (WBC), platelet count (PLT), neutrophil count (NEUT), mean corpuscular hemoglobin concentration (MCHC), serum creatinine (SCr), urea (UR), UA, fasting blood glucose (FBG), total cholesterol (TC), triglyceride (TG), total protein (TP), Alb, globulin (Glob), alpha fetoprotein (AFP), carcinoembryonic antigen (CEA), and alanine aminotransferase (Alt). All participants were required to confirm that they had fasted for at least 8 h and had venous blood drawn for testing, and all blood indicators were standardized and measured by hospital laboratory doctors.

### Definitions

In line with recommendations from the Kidney Disease Outcomes Quality Initiative, CKD can be assessed by decreasing the estimated glomerular filtration rate (eGFR). The CKD group was defined as stages 3 to 5 as calculated by the Modification of Diet in Renal Disease equation (eGFR less than 60 ml/min per 1.73 m^2^). The type and severity of disability are based on the classification and grading criteria of disability (GB/T 26341–2010) and the International Classification of Functioning, Disability, and Health. Physical disability is defined as a structural or functional injury to the human motor system resulting in the mutilation of the extremities or paralysis or deformity of the extremities or trunk resulting in varying degrees of loss of human motor function and limitations of activity or participation. BP and all blood biochemistry indicators were classified as normal or abnormal according to the normal reference range (Table S1).

### Statistical analysis

Statistical software R (version 4.0.2) was used for data management and analyses. All continuous variables conformed to a normal distribution according to the Kolmogorov‒Smirnov test. The T test and chi-squared test were used to analyse the crude difference between the CKD and non-CKD groups among 2679 subjects. Bar plots and line plots were used to depict trends in the number and prevalence of CKD in different age subgroups, respectively. Multiple logistic regression was used to assess the independent factors associated with the risk of CKD. Multivariable-adjusted odds ratios (AORs) and 95% confidence intervals (CIs) were calculated for each risk factor, indicating the magnitude of CKD risk. Forest plots were used to illustrate differences in independent risk factors for the risk of developing CKD between different disability subgroups. A two-sided p value less than 0.05 was considered statistically significant.

## Results

**Fig. 2:**
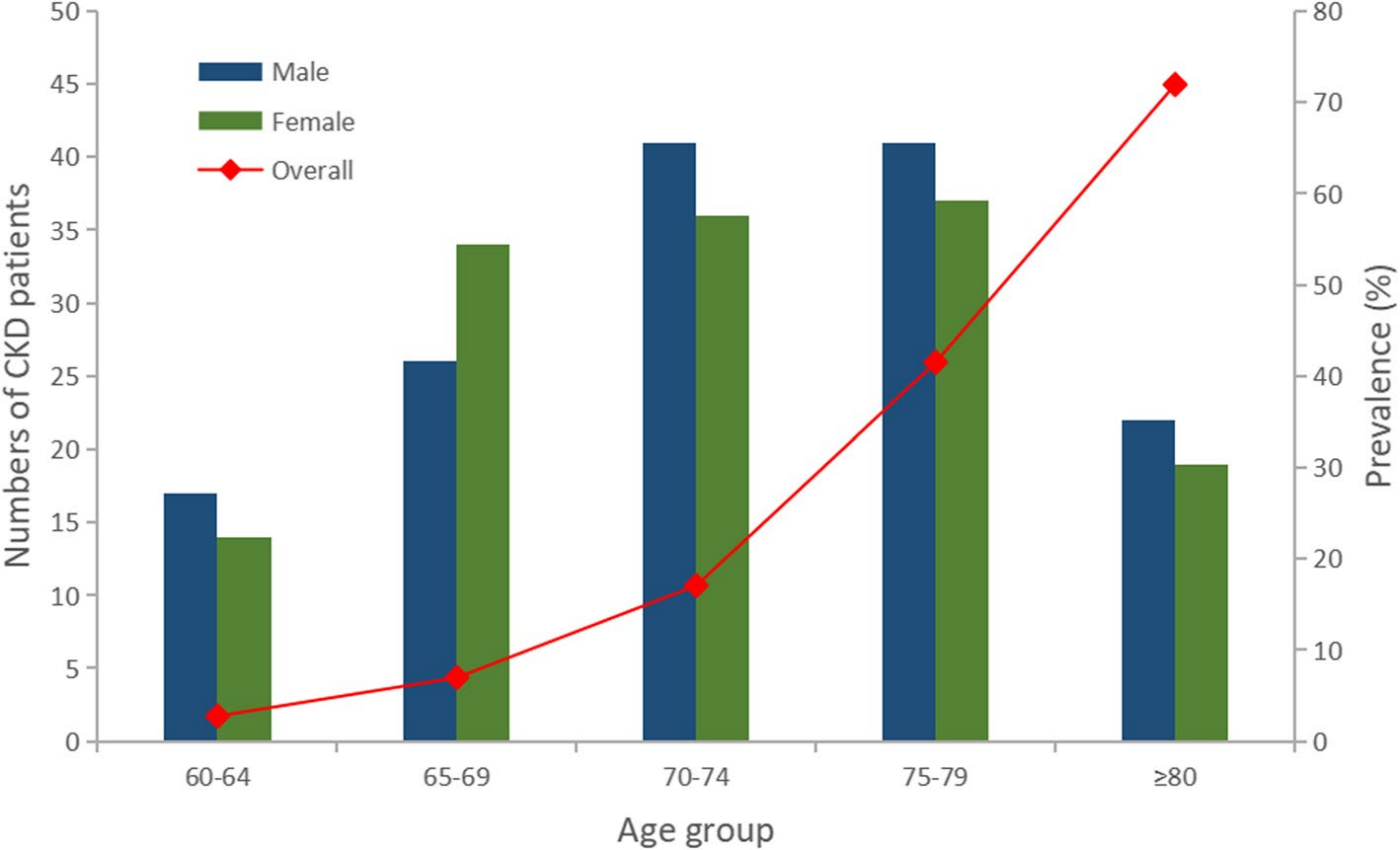
Prevalence of CKD in the study population by age and sex group

A total of 2679 participants with physical disabilities were included in this study, of which 287 (10.7%) participants with eGFR < 60 ml/min per 1.73 m^2^ were considered to have CKD. The prevalence was further calculated for each 5-year subgroup from age 60 onwards and is shown in Fig. 2. The prevalence of CKD increases gradually with age, from 2.7% in the 60–64 age group to a high of 71.9% in the over 80-year group.

Demographic and health-related characteristics of the study population by CKD status are summarized in Table 1. The mean values of BP and blood biochemistry among the study population are shown in Table S2. Of the 2679 participants, 58.0% were male, and the average age was 67.0 ± 5.3. The majority of participants had III-IV level disability (90.4%) and a middle school education (56.1%). The three most prevalent self-reported diseases were hypertension (55.4%), dyslipidemia (27.7%), and diabetes (15.3%). When comparing participants with CKD to those without CKD, there were significant differences in sex, age, education, BMI, history of hypertension, and levels of RBC, TG, Alb, CEA, Alt, UR, and UA (all *P* < 0.05).

**Table 1:** Baseline characteristics of study population with CKD group and Non-CKD group

Variables	Non-CKD group (*n* = 2392)	CKD group (*n* = 287)	*P*-value
**Sociodemographic features**			
Gender (male, %)	1406 (58.8)	147 (51.2)	0.014
Age (year)	66.28 ± 4.57	73.17 ± 6.47	< 0.001
Education (%)			< 0.001
Elementary school or less	465 (19.5)	113 (39.4)	
Middle school	1380 (57.7)	124 (43.2)	
At least some high school	547 (22.9)	50 (17.4)	
Level of disability (%)			0.160
I-II	237 (9.9)	21 (7.3)	
III-IV	2155 (90.1)	266 (92.7)	
SBP (Abnormal, %)	672 (28.1)	78 (27.2)	0.744
DBP (Abnormal, %)	119 (5.0)	13 (4.5)	0.742
BMI (kg^a^/m^2^)	24.72 ± 3.31	22.67 ± 3.17	< 0.001
**Medical history**			
Hypertension (yes, %)	1310 (54.8)	175 (61.0)	0.046
Diabetes (yes, %)	368 (15.4)	43 (15.0)	0.858
Dyslipidemia (yes, %)	667 (27.9)	75 (26.1)	0.531
Coronary heart disease (yes, %)	285 (11.9)	44 (15.3)	0.096
**Blood biochemistry**			
RBC (Abnormal, %)	199 (8.3)	36 (12.5)	0.017
WBC (Abnormal, %)	154 (6.4)	23 (8.0)	0.310
PLT (Abnormal, %)	236 (9.9)	21 (7.3)	0.166
NEUT (Abnormal, %)	111 (5.3)	20 (8.0)	0.075
MCHC (Abnormal, %)	337 (14.1)	37 (12.9)	0.580
FBG (Abnormal, %)	494 (20.7)	49 (17.1)	0.154
TC (Abnormal, %)	670 (28.0)	83 (28.9)	0.746
TG (Abnormal, %)	982 (41.1)	91 (31.7)	0.002
TP (Abnormal, %)	162 (6.8)	15 (5.2)	0.319
Alb (Abnormal, %)	132 (5.5)	37 (12.9)	< 0.001
Glob (Abnormal, %)	1113 (46.5)	143 (49.8)	0.290
A/G (Abnormal, %)	1571 (65.7)	203 (70.7)	0.087
AFP (Abnormal, %)	1 (0.0)	0 (0.0)	0.732
CEA (Abnormal, %)	139 (5.8)	29 (10.1)	0.005
ALT (Abnormal, %)	185 (7.7)	9 (3.1)	0.005
UR (Abnormal, %)	164 ( 6.9)	66 (23.0)	< 0.001
UA (Abnormal, %)	415 (17.3)	88 (30.7)	< 0.001

*SBP* Systolic Blood Pressure, *DBP* Diastole Blood Pressure, *BMI* Body Mass Index, *RBC* Red blood cell count, *WBC* White blood cell count, *PLT* Platelet, *NEUT* Neutrophil count, *MCHC* Mean corpuscular hemoglobin concentration, *FBG* Fasting blood glucose, *TC* Total cholesterol, *TG* Triglyceride, *TP* Total protein, *Alb* Albumin, *Glob* Globulin, *A/G* Albumin/ Globulin, *AFP* Alpha fetoprotein, *CEA* Carcinoembryonic antigen, *ALT* Alanine aminotransferase, *UR* Urea, *UA* Uric Acid

^a^The t-test and chi-squared test were used to analyze the crude difference between two groups

Multiple logistic regression was built to further analyse the adjusted association between potential influencing factors correlated with CKD (variables with *P* < 0.1 in Table 1). However, BMI was excluded from the analysis due to measurement bias, and a history of diabetes was included because it was an important cause of CKD. As displayed in Table 2, sex, age, history of hypertension, and levels of RBC, Alb, UR, and UA were independently associated with CKD. After adjusting for sex, age, and history of hypertension, abnormalities in RBC (AOR: 1.85, 95% CI: 1.11, 2.99), Alb (AOR: 1.77, 95% CI: 1.06, 2.89), UR (AOR: 4.18, 95% CI: 2.82, 6.20), and UA (AOR: 2.48, 95% CI: 1.77, 3.47) were significantly correlated with an increased risk of CKD.

**Table 2:** Multiple logistic regression analysis of the risk factors of CKD among 2679 physical disabilities

Predictors	Beta	AOR(95%CI)	*P*-value
Gender (female)	0.49	1.63(1.21,2.19)	0.001
Age (year)	0.22	1.25(1.21,1.28)	< 0.001
Education			
Elementary school or less	Reference	-	-
Middle school	-0.23	0.80(0.40,1.58)	0.513
At least some high school	-0.60	0.55(0.28,1.08)	0.082
Hypertension (yes)	0.29	1.33(1.00,1.80)	0.048
Diabetes (yes)	0.17	1.18(0.78,1.79)	0.425
Coronary heart disease (yes)	-0.40	0.96(0.64,1.44)	0.847
RBC (abnormal)	1.28	3.62(2.27,5.78)	< 0.001
NEUT (abnormal)	0.211	1.24(0.87,1.76)	0.255
TG (abnormal)	-0.04	0.96(0.17,5.43)	0.965
Alb (abnormal)	0.44	1.55(1.14,2.12)	0.006
A/G (abnormal)	-0.25	0.78(0.19,3.22)	0.729
CEA (abnormal)	-0.18	0.84(0.57,1.24)	0.382
ALT (abnormal)	-0.04	0.96(0.58,1.58)	0.886
UR (abnormal)	1.43	4.18(2.82,6.20)	< 0.001
UA (abnormal)	0.91	2.48(1.77,3.47)	< 0.001

*AOR* Adjusted Odd Ratio, *BMI* Body Mass Index, *RBC* Red blood cell count, *NEUT* Neutrophil count, *TG* Triglyceride, *Alb* Albumin, *A/G* Albumin/ Globulin, *CEA* Carcinoembryonic antigen, *ALT* Alanine aminotransferase, *UR* Urea, *UA* Uric Acid

**Fig. 3:**
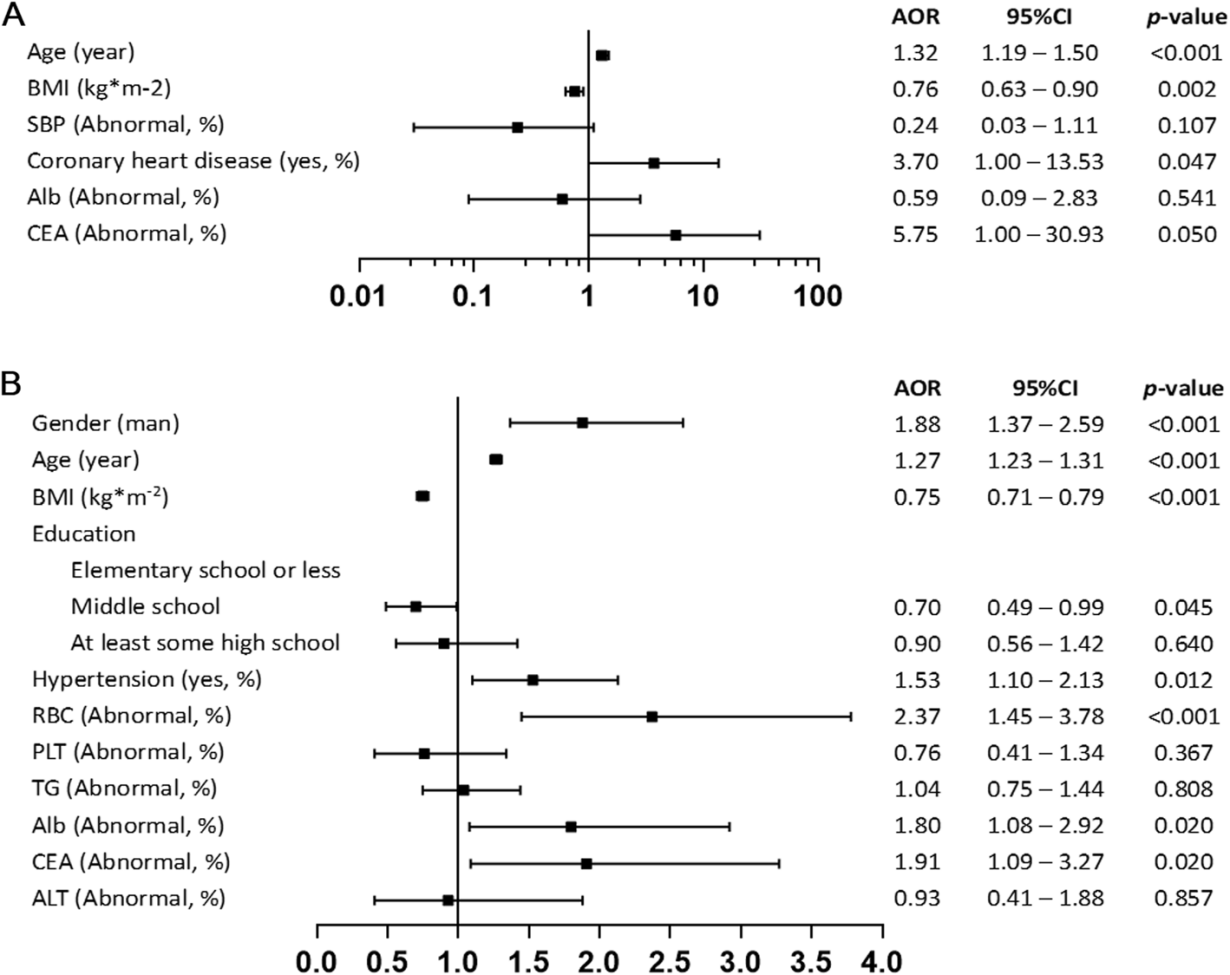
Subgroup analysis of CDK independent risk factors for different disability levels. **A** Grade I-II disability; **B** Grade III-IV disability

The prevalence of CKD in the severe (I-II) disability study population was 8.1%, and the prevalence in the mild (III-IV) disability study population was 11.0%. We conducted subgroup analyses according to different disability levels, and the baseline characteristics of the severe (I-II) and mild (III-IV) disability study populations are shown in Tables S3 and S4, respectively. All potential influencing factors were included in the logistic regression models separately, and the results are shown in Tables S5, S6, and Fig. 3. BMI was also excluded, and a history of diabetes was included. For the participants with severe disability, age (AOR: 1.31, 95% CI: 1.17, 1.47), history of coronary heart disease (AOR: 6.45, 95% CI: 1.60, 25.64), and the level of UA (AOR: 5.81, 95% CI: 1.79, 18.82) significantly increased the risk of CKD. For mild disability participants, abnormalities in RBC (AOR: 3.30, 95% CI: 2.02, 5.39), Alb (AOR: 1.49, 95% CI: 1.08, 2.06), UR (AOR: 4.42, 95% CI: 2.93, 6.68) and UA (AOR: 2.30, 95% CI: 1.61, 3.28) were independent risk factors for CKD after adjusting for sex and age.

## Discussion

This large-scale cross-sectional study from SDHS provided an estimated prevalence of 10.7% for CKD (eGFR < 60 mL/min per 1.73 m^2^) in the physically disabled population aged ≥ 60 years, which is significantly higher than that of the general elderly population in China, southwestern Nicaragua, and the UK [9, 10, 24]. Elderly individuals with physical disabilities are likely more prone to weakness, malnutrition, and muscle atrophy than older normal people, and a large amount of epidemiological evidence suggests that these factors are associated with reduced renal function [25–28]. Our finding that the prevalence of elderly patients over 80 years old is much higher than in other groups is consistent with those surveys in the general elderly population [9, 29, 30].

Risk factors correlated with CKD, including sex, age, and history of hypertension, as well as abnormalities in RBC, Alb, UR, and UA, were identified in the elderly physically disabled population. The relationships between gender, age, and history of hypertension are similar to those of the general population of the elderly [9, 10, 29, 30]. Age and hypertension related to CKD have been well clarified [1, 29–31], and high BP accounted for the largest proportion of CKD burden in East Asia, eastern Europe, tropical Latin America, and western sub-Saharan Africa. Public health policy has a role in slowing the incidence rate of ESRD through appropriate treatment of CKD risk factors such as high systolic BP. Meanwhile, hormonal changes after menopause are responsible for the higher incidence of renal insufficiency in females than in males [1, 7, 8, 29]. In addition, an important manifestation of impaired renal function is decreased RBC production, and the frequency and severity of anemia usually increase in the advanced stages of CKD [32]. Interestingly, the link between Alb and CKD is controversial. Menon et al. believed that the possible mechanism of Alb as a predictor of all-cause mortality in CKD is that it acts as a marker (nutritional index) different from the inflammatory process (such as malnutrition) [11, 12]. However, other studies have suggested that hypoalbuminemia is simply a reflection of a negative acute-phase response and that serum albumin levels may be more indicative of underlying inflammation than nutritional status, especially in CKD [11, 33]. Similarly, Seki et al. hold that higher UR levels were associated with adverse renal outcomes independent of the eGFR [34]. The release of pressin and activation of the aldose reductase-fructokinase pathway caused by elevated serum osmolality may be associated with renal injury, and UR is one of the substances that influence calculated serum osmolality levels [35]. Furthermore, experimental evidence suggests that UA may be harmful in CKD patients by promoting increased inflammation and CKD progression [36]. This potential link between increased UA levels and CKD progression has been supported further by some epidemiological studies [13, 14, 37], but not all [15], which may be because older age of the population may interfere with the identification of UA levels as an influencing factor of CKD [36]. It is noteworthy that there is a measurement bias in BMI because the weight of the elderly with physical disabilities is the actual value, while the height is the estimated value of the normal person, causing the BMI to be generally lower than that of the corresponding population. In follow-up studies of people with disabilities, the waist-to-hip ratio or visceral fat index could be used instead of BMI. The differences between systolic BP as well as FBG and CKD were not found in this study because the medications taken before the BP and blood glucose measurements were not collected, which may affect whether the systolic BP and FBG were normal. There were no differences in the history of diabetes and dyslipidemia among elderly physical disabilities with or without CKD, which may be due to the high prevalence of diabetes and dyslipidemia in both groups. Furthermore, both CKD patients and elderly people with disabilities are more prone to develop chronic diseases due to metabolic disorders [38–40].

Subgroup analysis revealed that the prevalence of CKD in the severe (I-II) disability group was lower than that in the mild (III-IV) disability group, and the disease-related factors in severely disabled patients were less than those in mildly disabled patients. This is related to the health difference brought by the degree of disability, not that patients with severe disabilities are healthier. Severely disabled patients often have less physical activity, lower self-care ability, and higher economic burden, which results in fewer opportunities to receive medical examinations and shorter life expectancy [41]. In this way, severely disabled patients with multiple diseases have difficulty going out for examinations, which means severely disabled patients who voluntarily participated in our study are healthier. Independent risk factors for CKD in patients with mild disabilities were consistent with those in the overall population. Coronary heart disease was a unique independent influencing factor of disease in patients with severe disabilities. Hypertension is a risk factor for coronary heart disease, as well as CKD [42]. Patients with coronary heart disease often have a worse level of BP management and are more prone to CKD. With the acceleration of aging, elderly individuals with physical disabilities are more likely to have comorbidities with chronic diseases [43]. CKD is largely preventable and treatable, and early recognition through the implementation of appropriate screening and intervention strategies (scientific treatment and dietary intervention) has been shown to slow disease progression and improve clinical outcomes.

Our study also has several limitations. First, the prevalence of CKD may be slightly overestimated using single measurements, as in most related studies. Second, self-report-based estimates provide an inaccurate basis for scientific conclusions while accounting for the potential effects of education and medical history, and we are also unable to assess the lifestyle, psychological and cognitive status, and medication use of disabled patients with limited data. Finally, potential residual confounding factors could not be excluded, although we adjusted for all known covariates, and the cross-sectional study prevented us from determining the causal relationship between CKD and associated risk factors. Further prospective cohort studies should be conducted to better understand the effect of CKD on patients with disabilities.

## Conclusions

The prevalence of CKD is high in patients with elderly physical disabilities, especially among people over 80 years old. In addition to the immutable variables, including gender, age, and history of hypertension, indicators of RBC, Alb, UR, and UA should also be taken into account in elderly physical disabilities. The preventive strategies for patients with two levels of disability should have different focuses.

### Supplementary Information

**Additional file 1: Table S1.** Normal reference range of blood pressure and blood biochemistry indicators. **Table S2.** The mean value of blood pressure and blood biochemistry among study population with CKD group and Non-CKD group. **Table S3.** Baseline characteristics of I-II disability population with CKD group and Non-CKD group. **Table S4.** Baseline characteristics of III-IV disability population with CKD group and Non-CKD group. **Table S5.** Multiple logistic regression analysis of the risk factors of CKD among I-II disability population. **Table S6.** Multiple logistic regression analysis of the risk factors of CKD among III-IV disability population.

## Data Availability

The data that support the findings of this study are available from the corresponding author upon reasonable request.

## Abbreviations

CKD: Chronic kidney disease
ESRD: End-stage renal disease
Alb: Albumin
UA: Uric acid
SDHS: Shanghai Disability Health Survey
BP: Blood pressure
BMI: Body mass index
RBC: Red blood cell count
WBC: White blood cell count
PLT: Platelet
NEUT: Neutrophil count
MCHC: Mean corpuscular hemoglobin concentration
FBG: Fasting blood glucose
TC: Total cholesterol
TG: Triglyceride
TP: Total protein
Alb: Albumin
Glob: Globulin
A/G: Albumin/Globulin
AFP: Alpha fetoprotein
CEA: Carcinoembryonic antigen
ALT: Alanine aminotransferase
UR: Urea
SBP: Systolic blood pressure
DBP: Diastole blood pressure
eGFR: Estimated glomerular filtration rate
AOR: Adjusted odds ratio
CI: Confidence interval
