# Supplementary material for: Prevalence and associated risk factors for chronic kidney disease in the elderly physically disabled population in Shanghai, China: a cross-sectional study
Source: BMC Public Health. 2023 Oct 12;23:1987. doi: 10.1186/s12889-023-16455-4 (PMC10568763; doi:10.1186/s12889-023-16455-4)
Supplement: Supplementary file 1 — Additional file 1: Table S1. Normal reference range of blood pressure and blood biochemistry indicators. Table S2. The mean value of blood pressure and blood biochemistry among study population with CKD group and Non-CKD group. Table S3. Baseline characteristics of I-II disability population with CKD group and Non-CKD group. Table S4. Baseline characteristics of III-IV disability population with CKD group and Non-CKD group. Table S5. Multiple logistic regression analysis of the risk factors of CKD among I-II disability population. Table S6. Multiple logistic regression analysis of the risk factors of CKD among III-IV disability population. [file 12889_2023_16455_MOESM1_ESM.docx]

**Additional file 1**

**Table S1** Normal reference range of blood pressure and blood biochemistry indicators

| **Indicators** | **Unit** | **Male** | **Female** | **Reference** |
| --- | --- | --- | --- | --- |
| **Blood pressure** |  |  |  |  |
| SBP | mmHg | 90-140 | 90-140 | 1 |
| DBP | mmHg | 60-90 | 60-90 | 1 |
| **Blood biochemistry** |  |  |  |  |
| RBC | ×10^12^/L | 4.09-5.74 | 3.68-5.13 | 6 |
| WBC | ×10^9^/L | 3.97-9.15 | 3.69-9.16 | 6 |
| PLT | ×10^9^/L | 85-303 | 101-320 | 6 |
| NEUT | ×10^9^/L | 2.0-7.0 | 2.0-7.0 | 6 |
| MCHC | g/L | 131-172 | 113-151 | 6 |
| FBG | mmol/L | 3.9-6.1 | 3.9-6.1 | 2 |
| TC | mmol/L | 3.1-5.2 | 3.1-5.2 | 3 |
| TG | mmol/L | 0.56-1.7 | 0.56-1.7 | 3 |
| TP | g/L | 60-80 | 60-80 | 4 |
| Alb | g/L | 35-50 | 35-50 | 5 |
| Glob | g/L | 20-30 | 20-30 | 4 |
| A/G | - | 1-2.5 | 1-2.5 | 4 |
| AFP | ng/mL | 1.57-8.35 | 1.30-8.13 | 7 |
| CEA | ng/ml | 0.6-5.43 | 0.43-4.26 | 7 |
| Alt | U/L | 9-50 | 7-40 | 8 |
| UR | mmol/L | 3.60-9.51 | 3.35-8.89 | 9 |
| UA | μmol/L | 179.2-460.9 | 130.2-443.4 | 9 |

SBP: Systolic Blood Pressure; DBP: Diastole Blood Pressure; BMI: Body Mass Index; RBC: Red blood cell count; WBC: White blood cell count; PLT: Platelet; NEUT: Neutrophil count; MCHC: Mean corpuscular hemoglobin concentration; FBG: Fasting blood glucose; TC: Total cholesterol; TG: Triglyceride; TP: Total protein; Alb: Albumin; Glob: Globulin; A/G: Albumin/ Globulin; AFP: Alpha fetoprotein; CEA: Carcinoembryonic antigen; ALT: Alanine aminotransferase;UR:Urea;UA: Uric Acid.

**REFERENCES**

1. Hypertension Alliance (China), Hypertension Branch of China Association for the Promotion of Healthcare International Exchange, China Hypertension Prevention and Treatment Guidelines Revision Committee, et al. Guidelines for the prevention and treatment of hypertension in China (2018 revised edition) [J]. China Cardiovascular Journal, 2019, 24(1):33.
2. American Diabetes Association. 2. Classification and diagnosis of diabetes: standards of medical care in diabetes-2018. Diabetes Care. 2018;41(Suppl 1):S13–S27. doi:10.2337/dc18-S002
3. National Cholesterol Education Program (NCEP). Expert Panel on Detection, Evaluation, and Treatment of High Blood Cholesterol in Adults (Adult Treatment Panel III). Third Report of the National Cholesterol Education Program (NCEP) Expert Panel on Detection, Evaluation, and Treatment of High Blood Cholesterol in Adults (Adult Treatment Panel III) final report. Circulation. 2002;106(25):3143–3421.
4. Koerbin G, Sikaris K, Jones GRD, Flatman R, Tate JR; AACB Harmonization Committee for Common Reference Intervals. An update report on the harmonization of adult reference intervals in Australasia. Clin Chem Lab Med. 2018 Dec 19;57(1):38-41. doi: 10.1515/cclm-2017-0920. PMID: 29305566.
5. Coley-Grant D, Herbert M, Cornes MP, Barlow IM, Ford C, Gama R. The impact of change in albumin assay on reference intervals, prevalence of 'hypoalbuminaemia' and albumin prescriptions. Ann Clin Biochem. 2016 Jan;53(Pt 1):112-6. doi: 10.1177/0004563215599560. Epub 2015 Jul 20. PMID: 26195486.
6. Wu X, Zhao M, Pan B, Zhang J, Peng M, Wang L, Hao X, Huang X, Mu R, Guo W, Qiao R, Chen W, Jiang H, Ma Y, Shang H. Complete blood count reference intervals for healthy Han Chinese adults. PLoS One. 2015 Mar 13;10(3):e0119669. doi: 10.1371/journal.pone.0119669. PMID: 25769040; PMCID: PMC4358890.
7. Zhang GM, Guo XX, Ma XB, Zhang GM. Reference Intervals of Alpha-Fetoprotein and Carcinoembryonic Antigen in the Apparently Healthy Population. Med Sci Monit. 2016 Dec 12;22:4875-4880. doi: 10.12659/msm.901861. PMID: 27941709; PMCID: PMC5156556.
8. Shang H, Chen W-x, Pan B-s, et al. Reference intervals for common tests of liver function, electrolytes and blood cell analysis of Chinese adults. Chin J Lab Med. 2013;36(5):393–94.
9. Yang Y , Jiang H , Tang A , et al. Reference intervals for serum bilirubin, urea, and uric acid in healthy Chinese geriatric population[J]. Journal of Clinical Laboratory Analysis, 2017:e22318.

**Table S2** The mean value of blood pressure and blood biochemistry among study population with CKD group and Non-CKD group

| **Variables** | **Non-CKD group (n=2392)** | **CKD group (n=287)** | ***P*-value** |
| --- | --- | --- | --- |
| **Sociodemographic features** |  |  |  |
| SBP (mmHg) | 129.84±16.45 | 129.06±17.26 | 0.450 |
| DBP (mmHg) | 76.83±8.48 | 74.88±8.88 | <0.001 |
| **Blood biochemistry** |  |  |  |
| RBC (T/L) | 4.70±0.45 | 4.43±0.57 | <0.001 |
| WBC (g/L) | 6.58±1.67 | 6.59±2.63 | 0.987 |
| PLT (g/L) | 219.55±58.23 | 209.96±63.76 | 0.009 |
| NEUT (g/L) | 4.01±1.37 | 4.11±1.49 | 0.272 |
| MCHC (g/L) | 142.87±13.88 | 133.53±15.98 | <0.001 |
| FBG (mmol/L) | 5.75±1.64 | 5.57±1.80 | 0.079 |
| TC (mmol/L) | 5.12±1.00 | 5.06±1.08 | 0.354 |
| TG (mmol/L) | 1.70±1.16 | 1.52±0.96 | 0.012 |
| TP (g/L) | 73.51±4.15 | 72.82±4.45 | 0.008 |
| Alb (g/L) | 43.54±2.32 | 42.69±2.49 | <0.001 |
| Glob (g/L) | 29.94±3.76 | 30.13±3.63 | 0.413 |
| A/G | 1.49±0.43 | 1.44±0.19 | 0.048 |
| AFP (μg/L) | 4.16±6.84 | 3.69±1.70 | 0.267 |
| CEA (ng/ml) | 2.00±2.07 | 2.36±1.91 | 0.005 |
| ALT (μg/L) | 21.86±15.92 | 16.44±9.64 | <0.001 |
| UR (mmol/L) | 5.29±1.36 | 6.43±2.21 | <0.001 |
| UA (μmol/L) | 325.47±81.54 | 356.25±94.87 | <0.001 |

SBP: Systolic Blood Pressure; DBP: Diastole Blood Pressure; RBC: Red blood cell count; WBC: White blood cell count; PLT: Platelet; NEUT: Neutrophil count; MCHC: Mean corpuscular hemoglobin concentration; FBG: Fasting blood glucose; TC: Total cholesterol; TG: Triglyceride; TP: Total protein; Alb: Albumin; Glob: Globulin; A/G: Albumin/ Globulin; AFP: Alpha fetoprotein; CEA: Carcinoembryonic antigen; ALT: Alanine aminotransferase;UR:Urea; UA: Uric Acid. ***^*^****The t-test was used to analyze the crude difference between two groups.*

**Table S3** Baseline characteristics of I-II disability population with CKD group and Non-CKD group

| **Variables** | **Non-CKD group (n=237)** | **CKD group (n=21)** | ***P*-value** |
| --- | --- | --- | --- |
| **Sociodemographic features** |  |  |  |
| Gender (male, %) | 161 (67.9) | 14 (66.7) | 0.905 |
| Age (year) | 65.58±4.28 | 72.23±6.81 | <0.001 |
| Education |  |  | 0.161 |
| Elementary school or less | 35 (13.6) | 30 (12.7) |  |
| Middle school | 147 (57.0) | 134 (56.5) |  |
| At least some high school | 76 (29.5) | 73 (30.8) |  |
| SBP (abnormal, %) | 67 (28.3) | 2 (9.5) | 0.063 |
| DBP (abnormal, %) | 14 (5.9) | 0 (0.0) | 0.252 |
| BMI (kg*m^-2^) | 24.55±3.25 | 21.96±3.87 | 0.001 |
| **Medical history** |  |  |  |
| Hypertension (yes, %) | 107 (45.1) | 12 (57.1) | 0.291 |
| Diabetes (yes, %) | 35 (14.8) | 1 (4.8) | 0.205 |
| Dyslipidemia (yes, %) | 69 (29.1) | 5 (23.8) | 0.606 |
| Coronary heart disease (yes, %) | 32 (13.5) | 6 (28.6) | 0.062 |
| **Blood biochemistry** |  |  |  |
| RBC (abnormal, %) | 23 (9.7) | 2 (9.5) | 0.979 |
| WBC (abnormal, %) | 20 (8.4) | 2 (9.5) | 0.865 |
| PLT (abnormal, %) | 29 (12.2) | 4 (19.0) | 0.370 |
| NEUT (abnormal, %) | 9 (4.3) | 2 (11.8) | 0.169 |
| MCHC (abnormal, %) | 53 (22.4) | 2 (9.5) | 0.169 |
| FBG (abnormal, %) | 56 (23.6) | 2 (9.5) | 0.138 |
| TC (abnormal, %) | 70 (29.5) | 8 (38.1) | 0.413 |
| TG (abnormal, %) | 89 (37.6) | 5 (23.8) | 0.210 |
| TP (abnormal, %) | 14 (5.9) | 1 (4.8) | 0.830 |
| Alb (abnormal, %) | 16 (6.8) | 4 (19.0) | 0.043 |
| Glob (abnormal, %) | 124 (52.3) | 10 (47.6) | 0.679 |
| A/G (abnormal, %) | 166 (70.0) | 17 (81.0) | 0.291 |
| AFP (abnormal, %) | 0 (0.0) | 0 (0.0) | - |
| CEA (abnormal, %) | 9 (3.8) | 3 (14.3) | 0.029 |
| ALT (abnormal, %) | 22 (9.3) | 0 (0.0) | 0.144 |
| UR (abnormal, %) | 17 (7.2) | 4 (19.0) | 0.096 |
| UA (abnormal, %) | 10 (4.2) | 11 (52.3) | <0.001 |

SBP: Systolic Blood Pressure; DBP: Diastole Blood Pressure; BMI: Body Mass Index; RBC: Red blood cell count; WBC: White blood cell count; PLT: Platelet; NEUT: Neutrophil count; MCHC: Mean corpuscular hemoglobin concentration; FBG: Fasting blood glucose; TC: Total cholesterol; TG: Triglyceride; TP: Total protein; Alb: Albumin; Glob: Globulin; A/G: Albumin/ Globulin; AFP: Alpha fetoprotein; CEA: Carcinoembryonic antigen; ALT: Alanine aminotransferase;UR:Urea; UA: Uric Acid. ***^*^****The t-test and chi-squared test were used to analyze the crude difference between two groups.*

**Table S4** Baseline characteristics of III-IV disability population with CKD group and Non-CKD group

| **Variables** | **Non-CKD group (n=2155)** | **CKD group (n=266)** | ***P*-value** |
| --- | --- | --- | --- |
| **Sociodemographic features** |  |  |  |
| Gender (male, %) | 1245 (57.8) | 133 (50.0) | 0.016 |
| Age (year) | 66.35±4.60 | 73.25±6.45 | <0.001 |
| Education |  |  | <0.001 |
| Elementary school or less | 435 (20.2) | 108 (40.6) |  |
| Middle school | 1246 (57.8) | 111 (41.7) |  |
| At least some high school | 474 (22.0) | 47 (17.7) |  |
| SBP (abnormal, %) | 605 (28.1) | 76 (28.6) | 0.856 |
| DBP (abnormal, %) | 105 (4.9) | 13 (4.9) | 0.992 |
| BMI (kg*m^-2^) | 24.74±3.32 | 22.73±3.11 | <0.001 |
| **Medical history** |  |  |  |
| Hypertension (yes, %) | 975 (45.2) | 100 (37.6) | 0.018 |
| Diabetes (yes, %) | 333 (15.5) | 42 (15.8) | 0.886 |
| Dyslipidemia (yes, %) | 598 (27.7) | 70 (26.3) | 0.622 |
| Coronary heart disease (yes, %) | 253 (11.7) | 38 (14.3) | 0.228 |
| **Blood biochemistry** |  |  |  |
| RBC (abnormal, %) | 176 (8.2) | 34 (12.8) | 0.012 |
| WBC (abnormal, %) | 134 (6.2) | 21 (7.9) | 0.292 |
| PLT (abnormal, %) | 207 (9.6) | 17 (6.4) | 0.088 |
| NEUT (abnormal, %) | 102 (5.4) | 18 (7.7) | 0.142 |
| MCHC (abnormal, %) | 284 (13.2) | 35 (13.2) | 0.992 |
| FBG (abnormal, %) | 438 (20.3) | 47 (17.7) | 0.307 |
| TC (abnormal, %) | 600 (27.8) | 75 (28.2) | 0.904 |
| TG (abnormal, %) | 893 (41.4) | 86 (32.3) | 0.004 |
| TP (abnormal, %) | 148 (6.9) | 14 (5.3) | 0.323 |
| Alb (abnormal, %) | 116 (5.4) | 33 (12.4) | <0.001 |
| Glob (abnormal, %) | 989 (45.9) | 133 (50.0) | 0.205 |
| A/G (abnormal, %) | 1405 (65.2) | 186 (69.9) | 0.125 |
| AFP (abnormal, %) | 1 (0.1) | 0 (0.0) | 0.728 |
| CEA (abnormal, %) | 130 (6.0) | 26 (9.8) | 0.019 |
| ALT (abnormal, %) | 163 (7.6) | 9 (3.4) | 0.012 |
| UR (abnormal, %) | 204 (9.5) | 62 (23.3) | <0.001 |
| UA (abnormal, %) | 189 (8.8) | 77 (28.9) | <0.001 |

SBP: Systolic Blood Pressure; DBP: Diastole Blood Pressure; BMI: Body Mass Index; RBC: Red blood cell count; WBC: White blood cell count; PLT: Platelet; NEUT: Neutrophil count; MCHC: Mean corpuscular hemoglobin concentration; FBG: Fasting blood glucose; TC: Total cholesterol; TG: Triglyceride; TP: Total protein; Alb: Albumin; Glob: Globulin; A/G: Albumin/ Globulin; AFP: Alpha fetoprotein; CEA: Carcinoembryonic antigen; ALT: Alanine aminotransferase;UR:Urea; UA: Uric Acid. ***^*^****The t-test and chi-squared test were used to analyze the crude difference between two groups.*

**Table S5** Multiple logistic regression analysis of the risk factors of CKD among I-II disability population

| **Predictors** | **Beta** | **AOR(95%CI)** | ***P*-value** |
| --- | --- | --- | --- |
| Age (year) | 0.27 | 1.31(1.17,1.47) | <0.001 |
| Gender (female) | -0.24 | 0.78(0.22,2.79) | 0.708 |
| SBP (abnormal) | -1.70 | 0.18(0.02,1.48) | 0.111 |
| Hypertension (yes) | 0.54 | 1.72(0.48,6.17) | 0.405 |
| Diabetes (yes) | 1.09 | 2.97(0.28,31.52) | 0.366 |
| Coronary heart disease (yes) | 1.86 | 6.45(1.60,25.64) | 0.009 |
| Alb (abnormal) | -0.22 | 0.80(0.01,52.06) | 0.917 |
| CEA (abnormal) | 1.18 | 3.24(0.87,12.03) | 0.079 |
| UA (abnormal) | 1.76 | 5.81(1.79,18.82) | 0.003 |

AOR: Adjusted Odd Ratio; BMI: Body Mass Index; SBP: Systolic Blood Pressure; Alb: Albumin; CEA: Carcinoembryonic antigen; UA: Uric Acid.

**Table S6** Multiple logistic regression analysis of the risk factors of CKD among III-IV disability population

| **Predictors** | **Beta** | **AOR(95%CI)** | ***P*-value** |
| --- | --- | --- | --- |
| Gender (female) | 0.55 | 1.73(1.27,2.36) | <0.001 |
| Age (year) | 0.22 | 1.25(1.21,1.28) | <0.001 |
| Education |  |  |  |
| Elementary school or less | Reference | - | - |
| Middle school | -0.20 | 0.92(0.41,1.66) | 0.579 |
| At least some high school | -0.59 | 0.55(0.28,1.10) | 0.093 |
| Hypertension (yes) | 0.20 | 1.23(0.89,1.68) | 0.208 |
| Diabetes (yes) | 0.07 | 1.08(0.71,1.63) | 0.717 |
| RBC (abnormal) | 1.19 | 3.30(2.02,5.39) | <0.001 |
| PLT (abnormal) | 0.03 | 1.03(0.56,1.92) | 0.917 |
| TG (abnormal) | -0.11 | 0.90(0.14,5.76) | 0.909 |
| Alb (abnormal) | 0.40 | 1.49(1.08,2.06) | 0.014 |
| CEA (abnormal) | -0.29 | 0.74(0.49,1.13) | 0.167 |
| ALT (abnormal) | -0.01 | 0.99(0.59,1.69) | 0.998 |
| UR (abnormal) | 1.49 | 4.42(2.93,6.68) | <0.001 |
| UA (abnormal) | 0.83 | 2.30(1.61,3.28) | <0.001 |

AOR: Adjusted Odd Ratio; BMI: Body Mass Index; RBC: Red blood cell count; PLT: Platelet; TG: Triglyceride; Alb: Albumin; CEA: Carcinoembryonic antigen; ALT: Alanine aminotransferase;UR:Urea; UA: Uric Acid.
